# Epidemiology and genotypes analysis of human papillomavirus infection in Huizhou, China

**DOI:** 10.3389/fpubh.2024.1440480

**Published:** 2024-08-09

**Authors:** Zhun Shu, Wenli Zhao, Xuebing Zhan, Jiaqi Zeng, Jingyi Li

**Affiliations:** ^1^Department of Pathology, Huizhou First Hospital, Huizhou, Guangdong, China; ^2^Department of Dermatology, Zhuhai People's Hospital (Zhuhai Clinical Medical College of Jinan University), Zhuhai, Guangdong, China; ^3^Department of Dermatology, Huizhou First Hospital, Huizhou, Guangdong, China

**Keywords:** human papillomavirus, cervical cancer, cervical lesions, genotype, infection, China

## Abstract

**Background:**

Human papillomavirus (HPV) is a main pathogenic factor for cervical carcinoma. The prevalence and genotypes distribution of HPV vary in different regions. Thus, our study aimed to investigate the epidemiology of HPV in Huizhou, China.

**Methods:**

HPV tests were detected in 5,325 female outpatients, we focused on the overall HPV prevalence, genotypes distribution, and the correlation of HPV genotypes with cervical cytology.

**Results:**

The overall HPV prevalence was 27.53%, HPV52, HPV58, HPV39, HPV16 and HPV51 were predominant genotypes with single infection rate of 70.80%. HPV infection rate showed a U-shaped age distribution, statistical differences were observed among 5 age groups (χ^2^ = 50.497, *p* < 0.01), and the higher positive rate was aged under 30 (34.42%) and above 60 (34.74%). Among high-risk HPV (hrHPV) infections, 60.69% involved NILM, 0.99% HSIL. The degrees of cervical lesions in multiple hrHPV infections were worse than those in single infection (*p* < 0.01).

**Conclusion:**

The HPV infection rate is high in Huizhou, Guangdong, single infection was predominant. HPV infection presented with a U-shaped age distribution. Multiple hrHPV infection was worrying since it may aggravate cervical lesions. Women should pay more attention to HPV detection and choose a more appropriate HPV vaccine according to local HPV type distribution.

## Introduction

Cervical cancer (CC) is one of the main causes of cancer death among women, the incidence of CC has risen by 10%–40% in young females over the last 30 years. In 2018, there were 570,000 cervical cancer cases and 311,000 deaths worldwide (1), while about 604,000 new cases and 342,000 mortalities were reported worldwide in 2020 (2). CC is the second most common malignant tumor affecting Chinese women (3). According to data reported by the International Agency for Research on Cancer in 2020, the incidence and mortality rates of CC were increased in China during 2000–2016, with 109,741 new cases and 59,060 new deaths (4). Human papillomavirus (HPV) is a common virus of sexually transmitted infection, and it is a main pathogenic factor for cervical intraepithelial neoplasia (CIN) and invasive CC (5, 6). HPVs are classified as high-risk HPV (hrHPV) including HPV16, 18, 31, 33, 35, 39, 45, 51, 52, 56, 58, 68 and 59; probable high-risk HPV (phrHPV) including HPV26, 53, 66, 70, 73, and 82; and low-risk HPV (lrHPV) containing HPV6, 11, 40, 42, 43, 44, 54, 61, 70, 72, 81, and CP6108, according to their association to carcinogenesis (7, 8). A persistent infection of high-risk HPV is strongly associated with the development of CC (1). The prevalence and genotypes distribution of HPV vary considerably and geographically among populations. The most common HPV types were 16, 18, 31, 33, 35, 45, 52 and 58 in the world (9), HPV 16, 58, 52, 33, 31 and 18 were the predominant genotypes in Chinese female (10). The five most common HPV types were HPV 16, 52, 58, 18 and 6 in the Pearl River Delta region of Guangdong province including Dongguan, Guangzhou, Huizhou, Jiangmen, Qingyuan, Shenzhen, Zhaoqing, Zhongshan, and Zhuhai (11). Huizhou, a Pearl River Delta region of Guangdong province, is situated in southeast China, there are currently no population-based studies on the epidemiology of HPV infection. Thus, we performed the following research to reveal the prevalence of HPV infection and genotypes in our region and develop intervention and prevention strategies to prevent related diseases caused by HPV.

## Materials and methods

### Population

#### Source population

All the female outpatients who received HPV tests from Huizhou First Hospital were source populations.

#### Study population

In this study, we enrolled 5,325 female outpatients (mean age, 35.67 ± 13.20 years) with HPV tests referred to the dermatology department of the Dermatology and Gynecology Huizhou First Hospital from August 2022 to June 2023 as our study population. All the female enrolled in this study should satisfy the following inclusion criteria before sampling: (1) had sexual life history; (2) no sexual intercourse in 24 h; (3) no vaginal cleaning and intravaginal medication in 72 h; (4) was not in the menstruation and pregnancy. The exclusion criterion was incomplete identity information. The study population satisfy all of the inclusion criteria and the exclusion criterion. The study was conducted in accordance with the Declaration of Helsinki, and approved by the Ethics Committee of Huizhou First Hospital. As a retrospective study, ethical registration is not mandatory for this study.

### Sample collection

Gynecologists and dermatologist collected cervical exfoliated cell samples from outpatients via cytobrush scoured the cervix uteri through clockwise 5 rotations, and stored the samples in a cell preservation solution. The samples were transfered with portable thermostats (2°C–8°C) and stored at the refrigerator (2°C–8°C) until HPV DNA extraction and genotyping could be performed.

### DNA extraction

1 mL liquid-based cytology specimen was added into a 1.5 mL centrifugal tube, centrifuged at 12,000 × *g* for 5 min, then the supernatant was discarded. The cell precipitation was suspended with 500 μL 1 × PBS buffer, centrifuged at 12,000 × *g* for 5 min, then the supernatant was discarded. Add 200 μL 1 × PBS to resuspend cell precipitation, DNA extraction was performed by an automated nucleic acid extraction instrument (A-32mini, HEALTH GENETECH, Ltd., Ningbo, China) using a pre-packed nucleic acid extraction kit (HEALTH GENETECH, Ltd., Ningbo, China).

### PCR amplification

The specific primers were designed according to the early expression genes (E6, E7 and E1) of HPV genome. Specific amplification and capillary electrophoresis separation of HPV genes was performed. The genes of different HPV types were identified by the length of specific amplification fragment.

Cervical exfoliated samples were tested for HPV DNA using HPV nucleic acid detection and genotyping kit (HEALTH GENETECH, Ltd., Ningbo, GuoXieZhuZhun 20,173,403,364). 25 HPV types (hrHPV): (HPV16, 18, 31, 33, 35, 39, 45, 51, 52, 56, 58, 59, 68; (phrHPV): 26, 53, 66, 73, 82; (lrHPV): 6, 11, 42, 43, 44, 81, 83) were identified by real-time fluorescence PCR. All operations were in strict accordance with kit instructions. The amplification reagent was configured according to 9 μL PCR premixed solution, 2 μL thermus aquaticus (Taq) and DNA 9 μL per sample. Amplification was started by 42°C for 5 min, initial denaturation at 94°C for 8 min, denaturation at 94°C for 30 s, annealing at 60°C for 30 s, elongation at 70°C for 1 min. In total, 35 cycles were performed. Finally, elongation at 70°C for 1 min and the PCR product is stored at 4°C. 1 μL PCR product and 9 μL miscible liquids (Hi-Di and fluorescent internal standard dyes) were added into 96-well microtiter plates for each well. The positive and negative control assays were done simultaneously. The specimen was centrifuged at 3,000 rpm for 1 min. And then denaturation at 84°C for 4 min in MultiGene Mini PCR. Finally, the reaction product was detected by a 3500Dx analyzer instrument (HEALTH GENETECH, Ltd., Ningbo, China).

### ThinPrep cytology test

Cytological pathology results were assessed by senior physicians, and classified as follows: negative for intraepithelial lesion or malignancy (NILM), atypical squamous cells of undetermined significance (ASC-US), low-grade squamous intraepithelial lesion (LSIL), atypical squamous cells-cannot exclude HSIL (ASC-H), and high-grade squamous intraepithelial lesion (HSIL).

### Statistical methods

The χ^2^ test or Fisher’s exact test was performed to analyze the positivity rates of variables among groups. All statistical analyses were performed using SPSS 20.0 (*p* < 0.05 indicated statistical significance).

## Results

### Overall epidemiology of HPV

**Figure 1:**
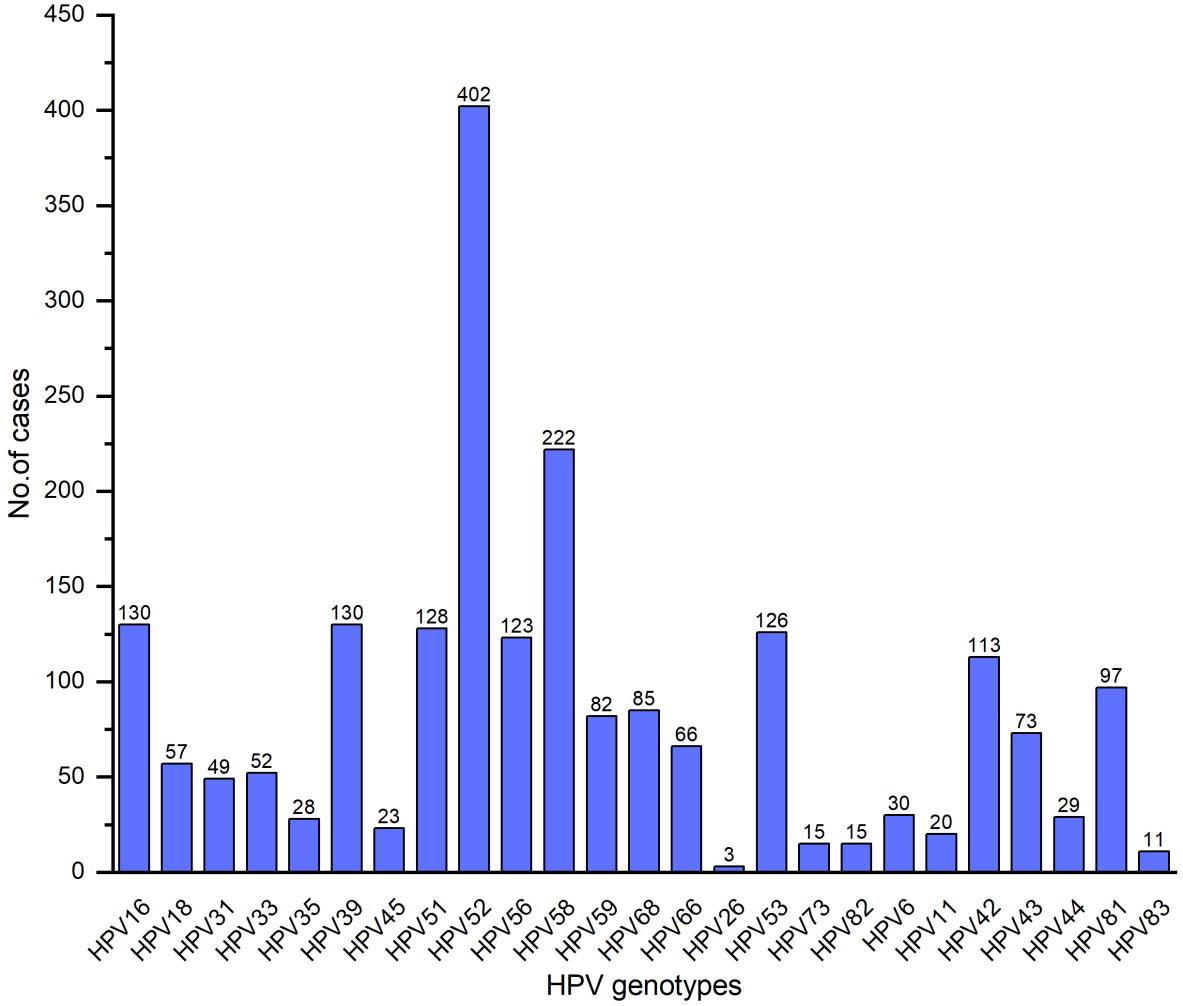
Various HPV genotypes distribution in patients.

5,325 patients underwent HPV tests wherein 1,466 (27.53%) patients were HPV positive. Of the 1,466 HPV-infected patients, 1,038 (70.80%) were single infection, 288 (19.65%) were dual infection, 93 (6.34%) were triple infection and 47 (3.21%) were more than triple infection. The subtypes of total 2,110 cases were detected, 1,511 (71.61%) hrHPV in which HPV52, HPV58, HPV39, HPV16 and HPV51 were predominant (Figure 1), 225 (10.66%) phrHPV and 374 (17.73%) lrHPV.

### Age distribution of HPV infection

**Figure 2:**
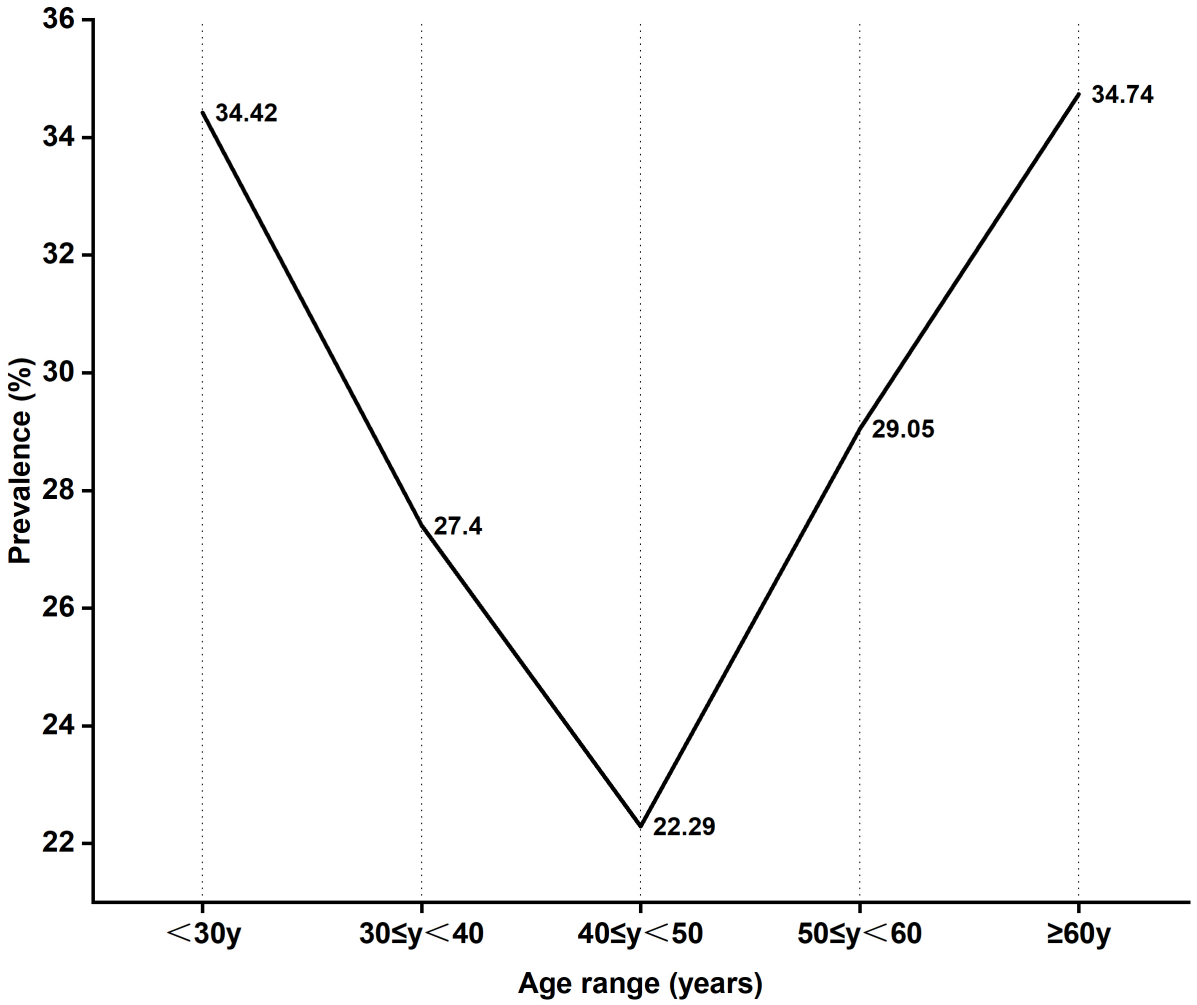
HPV infection presented with a bimodal age distribution.

Among the 5,325 patients who underwent HPV-tests, the age group 30–39 years was the highest number while those aged above 60 was the least number. The HPV infection rate of 5 age groups presented a U-shaped trend in which the highest positive rate of HPV infection was 34.42% in aged under 30 while the lowest positive rate was 22.29% in the age group 40–49 years. HPV-positive rate generally declined with age increasing in 30–50 years old females, the HPV-positive rate increased in ages above 50 (34.74%) (Figure 2). The prevalence of HPV infection was significantly different among the age groups (χ^2^, *p* < 0.01).

Single infection was predominant in all age groups. The distribution of HPV subtypes in different age groups is shown in Table 1.

**Table 1:** Distribution of HPV subtypes in different age groups.

HPV types	<30 y (*n* = 305)	30 ≤ y<40 (*n* = 473)	40 ≤ y<50 (*n* = 370)	50 ≤ y<60 (*n* = 244)	≥60 y (*n* = 74)
16	33(10.82)	409(86.47)	27(7.30)	22(9.02)	8(10.81)
18	11(3.61)	23(4.86)	14(3.78)	7(2.87)	2(2.70)
31	11(3.61)	19(4.02)	9(2.43)	8(3.28)	2(2.70)
33	15(4.92)	15(3.17)	10(2.70)	9(3.69)	3(4.05)
35	4(1.31)	10(2.11)	7(1.89)	2(0.82)	5(6.76)
39	28(9.18)	40(8.46)	34(9.19)	24(9.84)	4(5.41)
45	6(1.97)	10(2.11)	6(1.62)	1(0.41)	0
51	26(8.52)	38(8.03)	41(11.08)	15(6.15)	8(10.81)
52	96(31.48)	126(26.64)	94(25.41)	69(28.28)	17(22.97)
56	23(7.54)	40(8.46)	33(8.92)	21(8.61)	6(8.11)
58	49(16.07)	73(15.43)	49(13.24)	40(16.39)	11(14.86)
59	21(6.89)	24(5.07)	22(5.95)	14(5.74)	1(1.35)
68	16(5.25)	30(6.34)	14(3.78)	19(7.79)	6(8.11)
26	1(0.33)	1(0.21)	1(0.27)	0	0
53	28(9.18)	30(6.34)	30(8.11)	27(11.07)	11(14.86)
66	15(4.92)	24(5.07)	17(4.59)	8(3.28)	2(2.70)
73	0	7(1.48)	7(1.89)	0	1(1.35)
82	2(0.66)	7(1.48)	3(0.81)	2(0.82)	1(1.35)
6	92(30.16)	147(31.08)	100(27.03)	75(30.74)	23(31.08)
11	2(0.66)	6(1.27)	7(1.89)	4(1.64)	1(1.35)
42	20(6.56)	40(8.46)	28(7.57)	19(7.79)	6(8.11)
43	11(3.61)	27(5.71)	20(5.41)	15(6.15)	1(1.35)
44	9(2.95)	9(1.90)	2(0.54)	8(3.28)	1(1.35)
81	17(5.57)	30(6.34)	26(7.03)	19(7.79)	5(6.76)
83	2(0.66)	4(0.85)	2(0.54)	3(1.23)	0

### Cervical cytological status in high-risk genotypes infection

We found that hrHPV infections mainly involve NILM (60.69%), whereas ASC-US, ASC-H, LSIL and HSIL accounted for 21.71%, 1.99%, 5.56%, and 0.99%, respectively. The top five genotypes with NILM were HPV52 (26.94%), 58 (13.52%), 39 (9.81%), 56 (8.29%) and 16 (8.18%). The most prevalent HPV genotypes in HSIL were HPV52 (40.00%), 58 (26.67%), and 56 (20.00%). The degrees of cervical lesions in patients with multiple hrHPV infection were worse than those with single infection (*p* < 0.01), as LSIL and HSIL are more frequent in multiple hrHPV infections (Table 2).

**Table 2:** Cervical cytological status in high-risk genotypes infection.

	HSIL	LSIL	ASC-H	ASC-US	NILM	Undetected
	(*n* = 15)	(*n* = 84)	(*n* = 30)	(*n* = 328)	(*n* = 917)	(*n* = 137)
HPV 16	0	5(5.95)	4(13.33)	31(9.45)	75(8.18)	15(10.95)
HPV 18	1(6.67)	2(2.38)	0	13(3.96)	35(3.82)	6(3.65)
HPV 31	0	1(1.19)	0	9(2.74)	34(3.71)	5(3.65)
HPV 33	1(6.67)	2(2.38)	0	6(1.83)	37(4.03)	6(3.65)
HPV 35	0	1(1.19)	1(3.33)	6(1.83)	18(1.96)	2(1.46)
HPV 39	0	9(10.71)	1(3.33)	19(5.79)	90(9.81)	11(8.03)
HPV 45	0	2(2.38)	1(3.33)	5(1.52)	15(1.64)	0
HPV 51	0	10(11.90)	2(6.67)	28(8.54)	70(7.63)	18(13.14)
HPV 52	6(40.00)	23(27.38)	6(20.00)	91(27.74)	247(26.94)	29(21.17)
HPV 56	3(20.00)	5(5.95)	6(20.00)	24(7.32)	76(8.29)	9(6.57)
HPV 58	4(26.67)	14(16.67)	8(26.67)	54(16.46)	124(13.52)	18(13.14)
HPV 59	0	4(4.76)	0	16(4.88)	53(5.78)	9(6.57)
HPV 68	0	6(7.14)	1(3.33)	26(7.93)	43(4.69)	9(6.57)
shr-HPV	4(0.51)	31(3.92)	11(1.39)	44(5.57)	527(66.71)	59(7.47)
mhr-HPV	3(1.24)	12(4.96)	6(2.48)	50(20.66)	147(60.74)	24(9.92)

shr-HPV, single high-risk HPV; mhr-HPV, multiple high-risk HPV.

### Cervical cytological status in single HPV infection and multiple HPV infection

In our data, the single HPV infection for high-risk types, probable high-risk types and low-risk types were 790, 103 and 145 respectively, NILM was predominant while HSIL was less. In NILM, single HPV infection (54.23%) is more frequent than multiple HPV infections (45.77%). A similar situation prevails in ASC-H, ASC-US, LSIL and HSIL (Table 3).

**Table 3:** Cervical cytological status in single HPV infection and multiple HPV infection.

	shr-HPV (*n* = 790)	sphr-HPV (*n* = 103)	slr-HPV (*n* = 145)	s-HPV (*n* = 1,038)	m-HPV (*n* = 687)
HSIL	4(0.51)	1(0.97)	2(1.38)	10(58.82)	7(41.18)
LSIL	31(3.92)	11(10.68)	5(3.45)	66(75.86)	21(24.14)
ASC-H	11(1.39)	0	2(1.38)	19(67.86)	9(32.14)
ASC-US	158(20.00)	20(19.42)	28(19.31)	256(75.29)	84(24.71)
NILM	527(66.71)	62(60.19)	101(69.66)	615(54.23)	519(45.77)
Undetected	59(7.47)	6(5.83)	7(4.83)	72(60.50)	47(39.50)

shr-HPV, single high-risk HPV; sphr-HPV, single probable high-risk HPV; slr-HPV, single low-risk HPV; s-HPV, single HPV, m-HPV, multiple HPV.

## Discussion

Worldwide, 640,000 new cancer cases were attributable to HPV in 2012, inducing a significant threat to public health (12). According to the oncogenic potential, HPVs have been classified as hrHPV, phrHPV and lrHPV (7, 8), the lrHPVs cause benign lesions like condyloma acuminate and the hrHPVs are involved in the development and progression of CC, and have also been associated with cancers of the anus, penis, and head and neck (12). Persistent infection of hrHPV has been recognized as the necessary cause of CC (1). HPV prevalence and the distribution of HPV genotypes vary in different regions of the world which might be associated with education levels, socio-economic status, cervical screening and vaccination plan. The HPV infection rates are 7.3%–21.3% in China, 8.1% in Europe, 13.0% in the United States and 22.1% in Africa, respectively (13). In our study, the total HPV infection rate in Huizhou females was 27.53%, which was higher than that reported in Guangzhou (21.66%) (13), Luoyang (22.81%) (14), Xinjiang (14.02%) (15), Guangxi (18.10%) (16), Xiamen (18.4%) (17), but lower than in Fuzhou (38.3%) (18). The most common HPV types were 16, 18, 31, 33, 35, 45, 52 and 58 in the world (9); HPV 16, 18, 58, 52 and 51 in Africa; HPV 16, 53, 52, 18, and 39 in South, North and Central America; HPV 16, 52, 58, 18 and 56 in Asia; HPV 16, 18, 31, 33, and 58 in Europe (19). However, HPV 16, 58, 52, 33, 31, and 18 were the predominant genotypes in Chinese female (10). It is worth noting that there were also differences in the distribution of HPV infection genotypes in different regions in China. In the current study, HPV52, 58, 39, 16, and 51 were predominant. The five most common HPV types were HPV16, 52, 58, 18, and 6 in the Pearl River Delta region of Guangdong province (11). HPV 52, 16, 58, 51, 53, 39 and 68 were the common genotypes in Guangzhou (13); HPV 52, 16, 58, 51, 39, and 53 in Guangxi (16); HPV52, 58, 16, 51, and 39 in Xiamen (17); HPV 16, 52, 58, 18, and 51 in Luoyang Henan (14); HPV 16, 52, 58, 53, and 31 in Xinjiang (15). Analyzing the HPV genotype distributions in different regions is important for HPV vaccine development and developing effective prevention strategies for HPV.

Sexual activity and age are important determinants of HPV infection that are most common in sexually active females aged 18–30 years (20). Studies showed that HPV infection presented with a bimodal age distribution, the first age peak is 21–30 years old, and the second peak age is 51–60 years old (21). In our study, the higher positive rate was age under 30 and above 60. The reasons for the higher HPV-positive rate in females aged < 30 years may be related to sex hygiene, cervix immature and cervical epithelial more sensitive to carcinogens (22), however, in females aged≥60 years may be associated with a lower level of hormones and weaker ability to clear HPV (13, 22, 23). The reason for the decrease in incidence at age 40–50 may be they were infected in the past and HPV has been cleared by immunity system at the age of <30 so that the patients already have resistance to HPV infection. Therefore, self-protection is important before the peak age. HPV vaccination presents a primary prevention opportunity for the prevention of CC by achieving high levels of antibodies against carcinogenic HPV to prevent initial HPV infection (24). Currently, there are three prophylactic licensed HPV vaccines including a bivalent, a quadrivalent and a nine-valent, the nine-valent HPV vaccine could provide more comprehensive coverage. However, multivalent HPV vaccines were unable to cover all common HPV infection types and do not have therapeutic effects against pre-existing HPV infections (25). There were differences in the distribution of HPV genotypes in different regions, thus, local large-sample studies of HPV infections will be valuable in the development of HPV vaccines against CC in different regions.

In our study, NILM, LSIL and HSIL are more frequent in single infection, it could explain the higher incidents in single infection than multiple infections. Moreover, hrHPV infections is mainly involve in NILM (60.69%), whereas ASC-US, ASC-H, LSIL and HSIL accounted for 21.71%, 1.99%, 5.56%, and 0.99%, respectively. A similar situation prevails in single hrHPV, lrHPV, and phrHPV infection. It could be the reason for higher incidents in NILM than in LSIL and HSIL and the HPV infection in these patients with NILM may be temporary. Most HPV patients are transient infections, a small number of patients had persistent HPV infection, which further leads to cervical lesions (26). About 90% of HPV infections were resolved within 2–3 years by autoimmune or existed in a dormant state, approximately 10% were converted into chronic infection, and only 1% could cause CC (27). Also it can be positive for HPV in NILM, this could be because HPV DNA testing is more sensitive and specific than cytology (26). LSIL and HSIL are more frequent in multiple hrHPV infections than single hrHPV infection. It maybe because multiple HPV infections were associated with persistent HPV infection, and it is easier to clear HPV by immunity in a single infection than multiple infections (28). Persistent HPV infection, especially HPV 16 and 18, and lower immune response work together to LSIL, and then HSIL, and eventually progress to CC (5, 6). Cervical HPV infections, precancerous lesions and CC is an ongoing process and could extend about 20 years (29). Therefore, HPV detection is essential for the detection of high-grade lesions and the early diagnosis and prevention of CC. What’s more, HPV detection is more sensitive and can be used to detect CIN3 and CC combined with cytology, especially with higher sensitivity for CIN2/3 (30). Currently, cytology examination in combination with HPV detection is usually used for decreasing CC missed diagnoses. Our finding shows that the prevalence of HSIL in women with HPV 56 infection was higher than other HPV types, but higher LSIL in women with HPV 45 infection, which suggests that cervical diseases are closely related to HPV types. Studies have shown that cervical disease is closely associated with some factors such as HPV type, virus persistence, age and immune state (23), however, it remains unclear whether multiple HPV infection is a risk factor for persistent HPV infection and cervical lesions. Double HPV infection will greatly increase the risk of ASC-US, LSIL and HSIL (31). Several research suggested that multiple HPV types infection has a greater risk for developing CC than single HPV type infection (32). However, a different opinion implied that multiple HPV infection was not an increased risk factor of CC (33). In our study, LSIL and HSIL are observed more frequently in patients with multiple infection than single infection, which was similar to the research results of Luo et al. (23). However, the specific mechanism of multiple infection and co-infection will need further research.

In conclusion, the HPV infection rate is high in Huizhou, Guangdong, with HPV52, HPV58, HPV39, HPV16, and HPV51 as the common genotypes and single infection predominate. The age of HPV infection exhibits a U-shaped trend (<30 years old and >60 years old). LSIL and HSIL are observed more frequently in multiple HPV infections than single HPV infection. HPV typing detection is important and meaningful in the early detection and treatment of cervical lesions. Therefore, women should pay more attention to HPV genotypes detection, take preventive measures and choose a more appropriate HPV vaccine according to local HPV type distribution.

This study has several limitations. First, the data details are fairly limited, and HPV infection are not disaggregated by HPV vaccination status, sexual behavior, or occupation. Second, without the results of cervical histopathology, the association between multiple hrHPV infection and cervical lesions is speculative. Although we cannot obtain above details from existing data in a short time, we will still strive to collect relative data and further analyze the correlation between HPV infection and cervical lesions to optimize prevention measures in the future.

## Data availability statement

The raw data supporting the conclusions of this article will be made available by the authors, without undue reservation.

## Ethics statement

The studies involving humans were approved by the Ethics Committee of Huizhou First Hospital. The studies were conducted in accordance with the local legislation and institutional requirements. The ethics committee/institutional review board waived the requirement of written informed consent for participation from the participants or the participants’ legal guardians/next of kin because the study was conducted in accordance with the Declaration of Helsinki, and approved by the Ethics Committee of Huizhou First Hospital. Because the study was a retrospective study, confidential patient information were de-identified before the analysis and results were presented in aggregate level omitted subject identification. Therefore, the review committee waived the requirement for informed consent for this study.

## Author contributions

ZS: Data curation, Investigation, Writing – original draft, Formal analysis. WZ: Methodology, Writing – original draft, Resources. XZ: Software, Visualization, Writing – original draft. JZ: Conceptualization, Project administration, Writing – review & editing, Software. JL: Conceptualization, Project administration, Writing – review & editing, Supervision.
